# Overcoming Therapeutic Inertia in Multiple Sclerosis Care: A Pilot Randomized Trial Applying the Traffic Light System in Medical Education

**DOI:** 10.3389/fneur.2017.00430

**Published:** 2017-08-21

**Authors:** Gustavo Saposnik, Jorge Maurino, Angel P. Sempere, Maria A. Terzaghi, Christian C. Ruff, Muhammad Mamdani, Philippe N. Tobler, Xavier Montalban

**Affiliations:** ^1^Division of Neurology, Department of Medicine, St. Michael’s Hospital, University of Toronto, Toronto, ON, Canada; ^2^Laboratory for Social and Neural Systems Research, Department of Economics, University of Zurich, Zurich, Switzerland; ^3^Outcomes Research and Decision Neuroscience Unit, Li Ka Shing Knowledge Institute, St. Michael’s Hospital, University of Toronto, Toronto, ON, Canada; ^4^Neuroscience Area, Medical Department, Roche Farma, Madrid, Spain; ^5^Department of Neurology, Hospital General Universitario de Alicante, Alicante, Spain; ^6^Healthcare Analytics Research, Li Ka Shing Knowledge Institute, St. Michael’s Hospital, University of Toronto, Toronto, ON, Canada; ^7^Neurology-Neuroimmunology Department, Neurorehabilitation Unit, Multiple Sclerosis Centre of Catalonia (Cemcat), Barcelona, Spain

**Keywords:** multiple sclerosis, disease-modifying therapy, neuroeconomics, decision making, risk aversion

## Abstract

**Background:**

Physicians often do not initiate or intensify treatments when clearly warranted, a phenomenon known as therapeutic inertia (TI). Limited information is available on educational interventions to ameliorate knowledge-to-action gaps in TI.

**Objectives:**

To evaluate the feasibility and efficacy of an educational intervention compared to usual care among practicing neurologists caring for patients with multiple sclerosis (MS).

**Methods:**

We conducted a pilot double-blind, parallel-group, randomized clinical trial. Inclusion criteria included neurologists who are actively involved in managing MS patients. Participants were exposed to 20 simulated case-scenarios (10 cases at baseline, and 10 cases post-randomization to usual care vs. educational intervention) of relapsing–remitting MS with moderate or high risk of disease progression. The educational intervention employed a traffic light system (TLS) to facilitate decisions, allowing participants to easily recognize high-risk scenarios requiring treatment escalation. We also measured differences between blocks to invoke decision fatigue. The control group responded as they would do in their usual clinical practice not exposed to the educational intervention. The primary feasibility outcome was the proportion of participants who completed the study and the proportion of participants who correctly identified a high-risk case-scenario with the “red traffic light.” Secondary outcomes included decision fatigue (defined as an increment of TI in the second block of case-scenarios compared to the first block) and the efficacy of the educational intervention measured as a reduction in TI for MS treatment.

**Results:**

Of 30 neurologists invited to be part of the study, the participation rate was 83.3% (*n* = 25). Of the 25 participants, 14 were randomly assigned to the control group and 11 to the intervention group. TI was present in 72.0% of participants in at least one case scenario. For the primary feasibility outcome, the completion rate of the study was 100% (25/25 participants). Overall, 77.4% of participants correctly identified the “red traffic light” for clinical-scenarios with high risk of disease progression. Similarly, 86.4% of participants correctly identified the “yellow traffic light” for cases that would require a reassessment within 6–12 months. For the secondary fatigue outcome, within-group analysis showed a significant increased prevalence of TI in the second block of case-scenarios (decision fatigue) among participants randomized to the control group (TI pre-intervention 57.1% vs. TI post-intervention 71.4%; *p* = 0.015), but not in the active group (TI pre-intervention 54.6% vs. TI post-intervention 63.6%; *p* = 0.14). For the efficacy outcome, we found a non-significant reduction in TI for the targeted intervention compared to controls (22.6 vs. 33.9% post-intervention; OR 0.57; 95% CI 0.26–1.22).

**Conclusion:**

An educational intervention applying the TLS is feasible and shows some promising results in the identification of high-risk scenarios to reduce decision fatigue and TI. Larger studies are needed to determine the efficacy of the proposed educational intervention.

**Clinical Trial Registration:**

www.ClinicalTrials.gov, identifier NCT03134794.

## Background

Despite significant therapeutic advances, many patients remain undertreated, especially those with chronic medical conditions, such as atrial fibrillation, hypertension, and multiple sclerosis (MS) (1–4). One of the explanations relates to knowledge integration and knowledge-to-action gaps in therapeutic decisions. For example, it is known that physicians can be aware and informed about the current management of the common medical conditions they see in their daily clinical practice, but fail to integrate available information (e.g., severity of the condition, risk of progression, imaging findings, demographic factors affecting outcomes) and to implement best practice recommendations based on the available knowledge. This phenomenon may lead to therapeutic inertia (TI) usually associated with poorer outcomes (2–4). TI is a term that defines the absence of treatment initiation or intensification in patients when treatment goals are unmet. It affects 30–70% of clinicians caring for patients with chronic conditions (2, 5–7). Physician factors (e.g., low tolerance to uncertainty, *status quo* bias) are considered to be the main contributors to TI, but remain poorly studied (8–10).

Given physicians’ limited training in risk management and formal learning in medical decision-making, educational interventions could optimize medical decisions (11). Previous research suggests that such interventions can improve medical decisions. A meta-analysis comprising 609 eligible studies enrolling 35,226 trainees compared the efficacy of simulation-based educational interventions (e.g., case-scenarios) in clinical skills and medical decision-making. The authors showed that a simulation-based educational intervention was more effective than standard educational programs for outcomes of knowledge, skills, and trainee’s behavior (12). Other studies using a simulation-based intervention and clinical reasoning revealed a reduction in medical errors (13, 14). We have scarce information on strategies to overcome TI and only little evidence is available regarding effective educational interventions to reduce “knowledge-to-action” gaps.

The traffic light system (TLS) is an strategy that facilitates the decision-making process using traffic light terminology to match three types of situations: red light (“high risk”/“stop and think”), yellow light (warning), and green light (“stable”/“continue the same strategy”). The TLS emerged as a warning and risk categorization strategy to reduce human errors (15). It relies in a “hard-wired” cross-cultural color-coded concept that facilitates the integration of specific situations with an action (16–18). For example, studies showed that the TLS facilitated healthier food choices by interfering with automatic decisions and triggering re-evaluation processes (19). We focus on MS because of the broad availability of therapeutic options and clear definitions (clinical and radiological) of disease activity as the accepted criteria to escalate treatment.

We hypothesized that an educational intervention using the TLS may be feasible and effective to overcome insufficient knowledge integration and knowledge-to action gaps in the management of MS. In the present study, we evaluated the feasibility of an educational intervention to identify clinical situations of moderate and high risk of disease progression that may lead to TI. Our intervention was designed following the results of our previous studies on TI in MS care (4, 20).

## Methods

### Study Design and Participants

**Figure 1:**
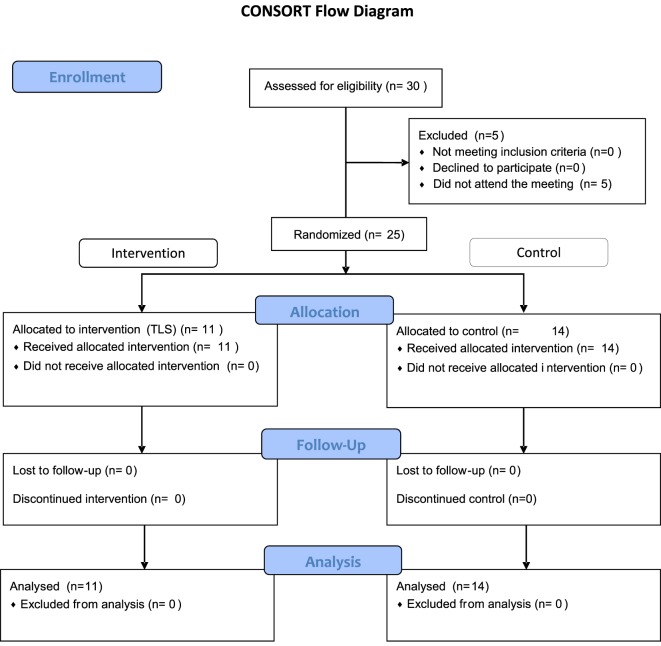
CONSORT flow diagram.

This pilot, double-blinded, parallel-group, randomized clinical trial evaluated the feasibility of an educational intervention (active group) compared to usual care (control group) in the management of MS (Figure 1—CONSORT flow diagram). The goal of the education intervention was to facilitate risk stratification-action gaps in MS care. We expected the TLS would facilitate the identification of high-risk clinical-scenarios (i.e., “red” in the TLS) leading to assertive therapeutic decisions (e.g., escalate therapy when appropriate). Inclusion criteria included neurologists who were actively involved in managing MS patients. Physicians whose practice was primarily in caring for MS patients were classified as “MS specialists.”

Candidates were invited to participate in a face-to-face meeting held in Madrid, Spain. The recruitment of participants was facilitated by the Spanish Neurological Society. We targeted the first 30 participants who replied to an e-mail invitation from a pool of neurologists who met the inclusion criteria. The study was conducted using Qualtrics, a web-based platform (www.Qualtrics.com). Each participant was provided with a tablet PC to complete the study. Participants were randomized (1:1 ratio), an automatic process in Qualtrics. Allocation concealment was facilitated in Qualtrics, so participants did not know what intervention will be allocated to after completing the 10 initial case-scenarios.

The study comprised 20 MS case-scenarios (see Appendix). Participants were exposed to 10 baselines case-scenarios (Block 1). Then, participants were randomized to usual care vs. educational intervention (TLS) followed by 10 additional similar case-scenarios (Block 2). In-line with the learning and education literature, case-vignettes, clinical scenarios, or “real world” encounters are regarded as the best simple strategy to evaluate cognitive biases among physicians (21, 22).

Case-scenarios were designed by our research team and MS experts (Angel P. Sempere, Gustavo Saposnik, Jorge Maurino, and Xavier Montalban). Overall, 16 cases were designed to assess appropriate escalation of treatment (absence of treatment escalation corresponding to TI; cases # 1–5, 8–10, 11–15, and 18–20), whereas the remaining four cases (case # 6, 7, 16, and 17) were designed to assess overtreatment (treatment escalation when there was no evidence of disease activity). Participants randomized to the intervention group (TLS) were also asked to identify the appropriate traffic light that would match the case-scenario. That question was prior to the selection of the therapeutic option.

**Figure 2:**
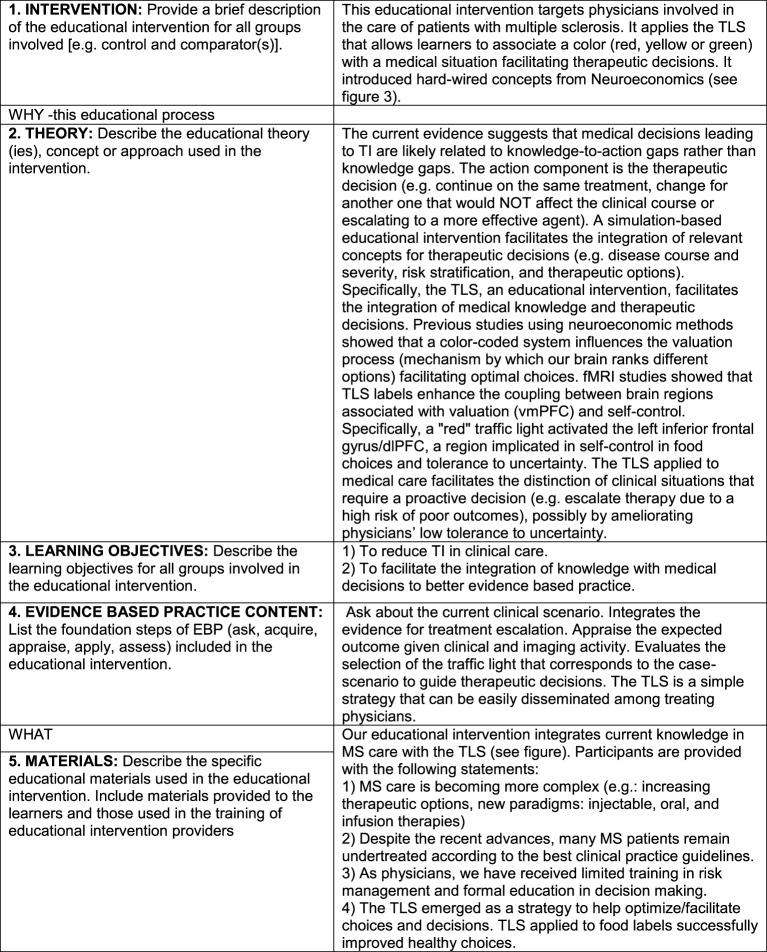

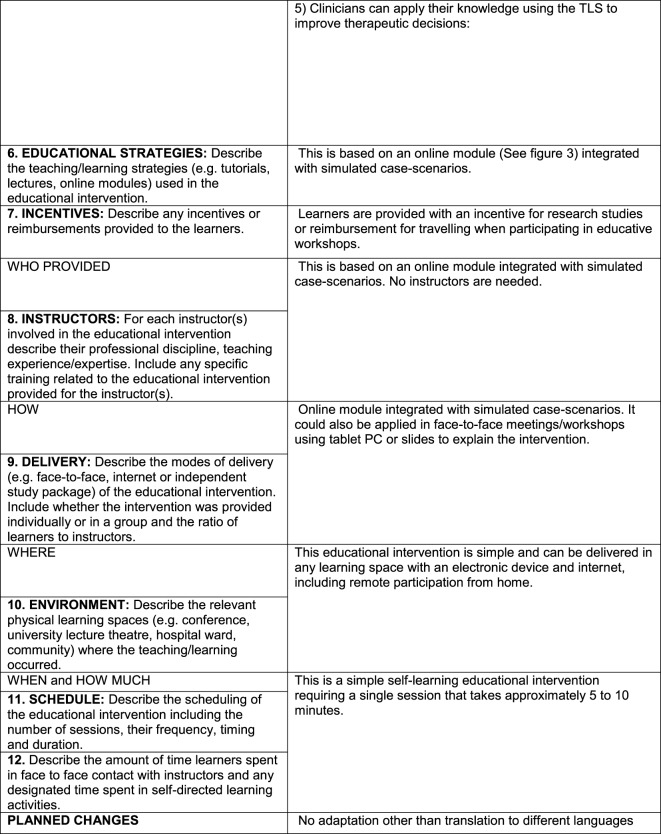

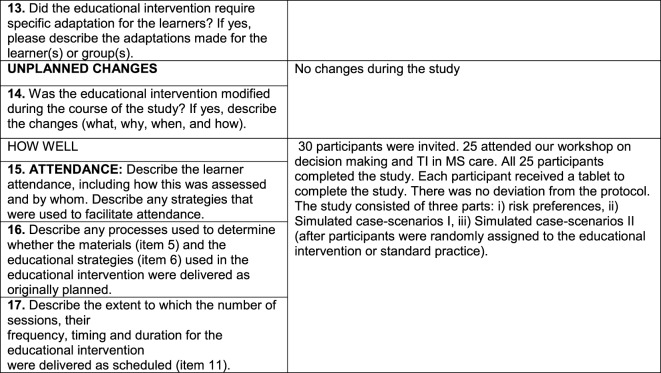
Description of the educational intervention according to the GREET guidelines.

**Figure 3:**
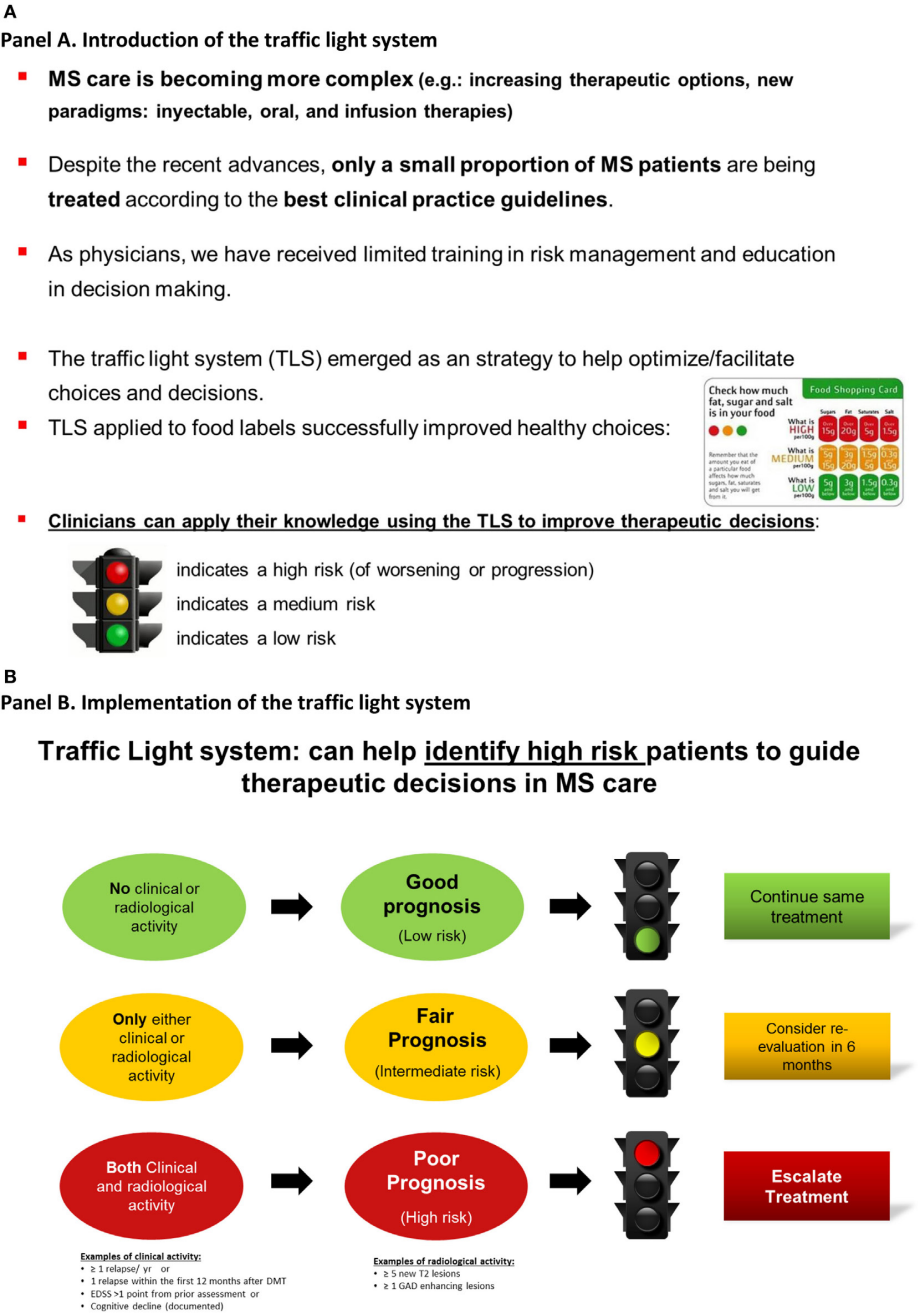

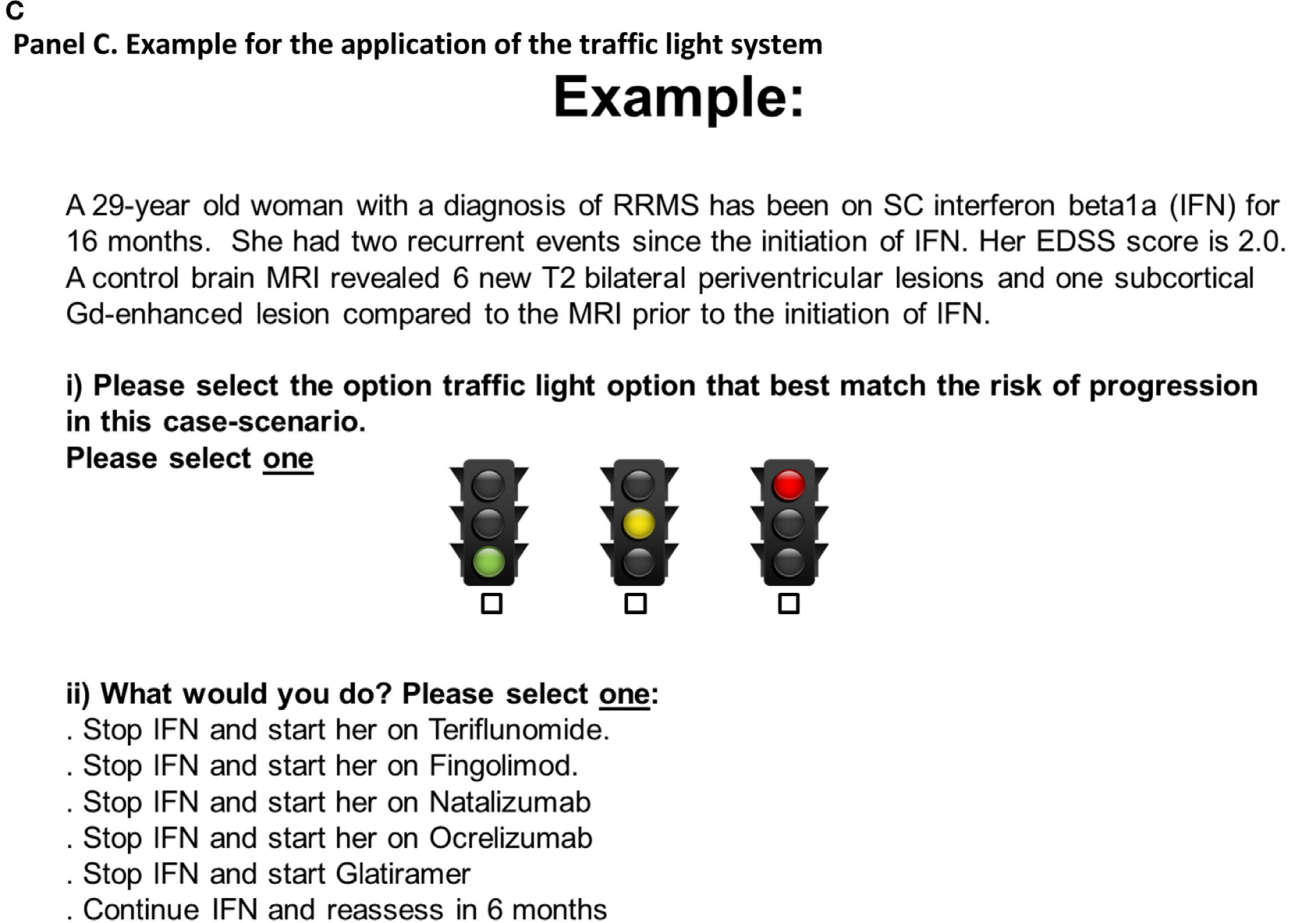
Educational intervention: the traffic light system may facilitate therapeutic decisions in multiple sclerosis care. Participants viewed the two informative panels **(A,B)** and a third panel providing an example **(C)**.

In Block 2, eight cases corresponded to clinical situations of high risk of MS progression, which participants in the intervention group should associate with the “red traffic light,” whereas two cases were associated with moderate risk of progression requiring a re-assessment in a 6- to 12-month period, which participants should associate with the “yellow traffic light” (see Figures 2 and 3).

Data management, research coordination, and statistical analyses were conducted at the Li Ka Shing Knowledge Institute of St. Michael’s Hospital, Toronto. Operational procedures, guidelines for the implementation of both arms of the study, and the consent form were approved by the ethics review board at St. Michael’s Hospital, University of Toronto. Online informed consent was obtained from all participants. The study was conducted in Spanish. Participants received compensation for transportation. Further details of the protocol were published in www.ClinicalTrials.gov # NCT03134794 and elsewhere (20).

### Rationale and Description of the Interventions

The current evidence suggests that medical decisions leading to TI are likely related to knowledge-to-action gaps (23, 24). We developed a simulation-based educational intervention aimed at facilitating the integration of knowledge and overcoming knowledge-to-action gaps in MS care (25). The *action* component is the *therapeutic decision* (e.g., continue on the same treatment, change to a treatment that would not affect the clinical course or escalating to a more effective agent). We followed the Guideline for Reporting of Evidence-based practice Educational interventions and Teaching (GREET) statement to describe our educational intervention (Figure 2) (26).

### Educational Intervention: The TLS

Our study included two phases: pre-intervention and post-intervention periods (Figure 3). Participants were randomly assigned to the educational intervention or control groups after the pre-intervention period.

Our educational intervention is based on the application of the TLS to medical decision-making (16–19, 27). In our study, the TLS was applied to help participants identify high-risk cases-scenarios, where MS patients had both clinical and radiological activity. Consequently, participants should be able to identify the “red” traffic light and escalate treatment. The “yellow” represents caution when MS patients had either a clinical relapse or some degree of activity on brain imaging (but not both), which requires a reassessment within 6–12 months.

The control group made therapeutic decisions without being exposed to the educational intervention as part of the current standard practice. They had the option to take a break or continue the study. The estimated time of study completion per participant ranged between 30 and 35 min and did not differ between groups.

#### Definitions

For the primary analysis, high risk of progression was defined as the combination of a clinical relapse plus the presence of new brain lesions in follow-up magnetic resonance imaging (MRI) scans or at least one gadolinium-enhancing lesion (28, 29). All high-risk simulated clinical cases included a description of an MRI with more than five new T2 lesions or at least one enhancing lesion (30). The use of these definitions combining a clinical relapse and MRI activity is consistent with recent evidence regarding the risk of treatment failure among patients receiving interferon-β (31). Disease progression was defined as at least one point worsening from baseline in the Expanded Disability Status Scale score (32).

Recent meta-analysis confirmed that alemtuzumab, natalizumab, and fingolimod are the best available choices for preventing clinical relapses in patients with relapsing–remitting MS (RRMS) (33). The current treatment option for RRMS include first-line (beta interferons, glatiramer acetate), second-line (fingolimod), and third-line (natalizumab, alemtuzumab) therapies. For the present analysis, we used the aforementioned scheme according to the current clinical practice (4, 34, 35).

### Outcome Measures

The primary feasibility outcome was the proportion of participants who completed the study. A completion rate of 70% or higher was our pre-specified outcome. The feasibility of delivering the intervention was defined as the number of participants who correctly identified the “red traffic light” for clinical-scenarios comprising a high-risk of progression. A pre-specified criterion of at least 70% correct responses was used to classify the educational intervention as feasible.

Efficacy of the educational intervention, a secondary outcome measure, was defined as a reduction in TI based on each individual response. We also evaluated secondary outcome the capability of the intervention to protect against decision fatigue decision fatigue [defined as the difference in TI within groups before (Block 1) and after the intervention (Block 2)] (36, 37). A significantly higher prevalence of TI in the 10 case-scenarios post-intervention (Block 2) would be indicative of decision fatigue.

### Statistical Analysis

Given the pilot nature of this study, we performed primarily descriptive statistics. We used non-parametric tests to compare continuous and categorical variables between groups. A Welch’s *t*-test was used to rule out large differences in TI. Logistic regression analysis was completed to determine the efficacy of the educational intervention in the reduction of TI after adjusting for responses in the pre-intervention period (Block 1).

We evaluated two different outcome measures: (i) TI defined as lack of treatment escalation in at least one case scenario and (ii) number of participants’ responses representing TI. We also compared the total number of correct responses for each case-scenario between and within groups before and after the intervention. Given our pre-specified target intervention, we compared the proportion of responses associated with TI between those who selected the “red light” in the active group vs. control group.

All tests were two-tailed, and *p*-values <0.05 were considered significant. We used STATA 13 (College Station, TX, USA: StataCorp LP) to conduct all analyses.

## Results

Of 30 neurologists from across Spain who were invited to participate in the study, 25 (83.3%) attended the meeting. Eleven participants were randomly assigned to the educational intervention, whereas the remaining 14 were assigned to the control group.

Overall, the mean (SD) age was 35.4 (±7.3) years; 16 (64%) were females. Sixty percent (15/25) of participants primarily focused their practice on MS care. Table 1 summarizes baseline characteristics of the study population. Baseline characteristics appeared similar between groups. None of the participants choose to have a break.

**Table 1:** Baseline characteristics of participants.

Characteristics	Total (%) *n* = 25	Intervention (%) *n* = 11	Control (%) *n* = 14
Age (mean ± SD), in years	35.4 ± 7.3	33.8 ± 5.5	36.6 ± 8.4
Sex			
Female	16 (64.0)	8 (72.7)	8 (57.1)
Specialty			
Multiple sclerosis (MS) specialists	15 (60.0)	6 (54.6)	9 (64.3)
General neurologists who care for MS patients	10 (40.0)	5 (45.5)	5 (35.7)
Practice setting			
Academic	22 (88.0)	11 (100)	11 (78.6)
Community	2 (8.0)	0 (0)	2 (14.3)
Both (academic and non-academic)	1 (4.0)	0 (0)	1 (7.1)
% time in clinical practice			
Greater than 75%	16 (64.0)	6 (54.6)	10 (71.4)
Years in practice, mean (±SD)	9.9 ± 7.3	8.5 ± 5.0	11.1 ± 8.7
MS patients seen per week, mean (±SD)	17 ± 11.0	15.9 ± 10.5	17.8 ± 11.7
Attended latest ECTRIMS conference	14 (56.0)	5 (45.5)	9 (64.3)
Author of a peer-reviewed publication in the last 12 months	12 (48.0)	3 (27.3)	9 (64.3)

*Numbers in brackets indicate percentages, unless otherwise indicated*.

The mean time to start the second set of cases post-randomization (block 2) was 12.7 s in the control group and 11.8 s in the intervention group.

For the primary feasibility outcome, the completion rate of the study was 100% (25/25 participants). TI was present in 72.0% of participants in at least one case scenario. Only 4 (16%) participants did not exhibit TI (all case-scenarios were correct), whereas one-third of participants (8/25) exhibited TI in five or more simulated case-scenarios. In the within-group analysis, we observed an increased prevalence of TI in the second set of case-scenarios (defined as decision fatigue) among participants in the control group (TI pre-intervention 57.1% vs. TI post-intervention 71.4%; *p* = 0.015), but not in the active group (TI pre-intervention 54.6% vs. TI post-intervention 63.6%; *p* = 0.14). Decision fatigue was associated with higher odds of TI (OR 3.99; 95% CI 1.05–15.1).

There was no TI block-by-intervention group interaction (*p* = 0.61). Comparative results between pre- and post-intervention within and between groups are summarized in Table 2.

**Table 2:** Efficacy outcome measures: comparison pre- and post-intervention within and between groups.

	Intervention group			Control group			Comparison between groups
Efficacy outcomes	Pre-intervention	Post-intervention	Change from pre-intervention (95% CI)^a^	Pre-intervention	Post-intervention	Change from pre-intervention (95% CI)^a^	Differences between groups post-intervention (95% CI)^b^
					
	*n* = 11	*n* = 11		*n* = 14	*n* = 14		
Mean (SD) number of individual responses related to therapeutic inertia (TI)	1.91 (1.3)	2.36 (1.5)	0.45 (−2.20, 1.31)	2.14 (1.4)	2.71 (1.8)	0.57 (−2.61, 1.48)	0.35 (−1.01, 1.71)
Individual responses related to TI, *n*/*N* (%)	21/88 (0.24)	26/88 (0.30)	0.056 (−1.30, 1.41)	30/112 (0.27)	38/112 (0.34)	0.071 (−1.18, 1.32)	0.044 (−1.33, 1.42)
% (SD) of participants with TI^c^	54.5 (27.2)	63.6 (25.4)	9.1 (−14.2, 32.4)	57.1 (26.4)	71.4 (22.0)	14.3 (−4.5, 33.1)	7.80 (−12.2, 27.8)

*n, number of responses related with TI; N, total number of responses. Numbers were rounded to two decimals*.

*^a^Represents the difference and 95% CI in the efficacy outcomes between pre- and post-intervention within groups*.

*^b^Represents the difference and 95% CI in the efficacy outcomes post-intervention between groups*.

*^c^TI identified in at least one case-scenario*.

Overall, 77.4% of participants correctly identified the “red traffic light” for clinical scenarios with high-risk of disease progression. Similarly, 86.4% of participants correctly identified the “yellow traffic light” for cases that would require a reassessment within 6–12 months. Thus, participants knew what should be done with different cases.

**Figure 4:**
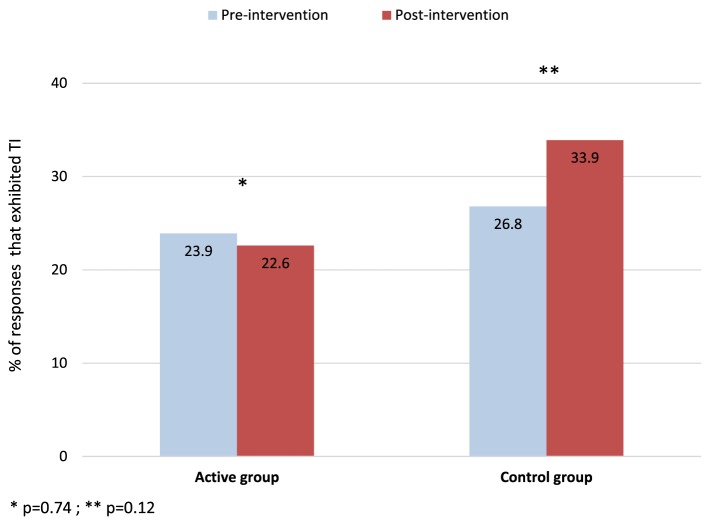
Prevalence of therapeutic inertia (TI) for the targeted intervention accounting for each individual response in the active and control groups. Lower numbers represent lower TI (more optimal therapeutic decisions). *p = 0.74, **p = 0.12. Note the lower trend in the prevalence of TI in the intervention group compared to the control group (22.6 vs. 33.9%; OR 0.57; 95% CI 0.26–1.22).

The analysis of each individual case-scenario revealed that TI was present in 23.9% of responses in the interventional group and 26.8% of responses in the control group in the pre-intervention period (*p* = 0.74) (Figure 4). The multivariate analysis of each individual response revealed a non-significant reduction in TI in favor of the intervention group (OR 0.82; 95% CI 0.25–2.69) after adjusting for pre-intervention TI. The analysis evaluating individual responses targeted by the intervention (those cases where participants correctly identified the red light for high-risk scenarios) revealed a non-significant reduction of TI in the intervention group compared to the control group (OR 0.57; 95% CI 0.26–1.22) (Figure 4).

## Discussion

Therapeutic inertia is a common phenomenon in the management of patients with chronic medical conditions (2, 8). Moreover, there is limited information regarding educational interventions to overcome the effects of TI. In the present study, we used the well-defined paradigm of MS care, with its broad variability of therapeutic options to escalate treatment as a response to evidence in disease activity.

We conducted a pilot randomized study allocating participating neurologists to an educational intervention (using the TLS) or usual care (control group). We found that TI was present in at least one case-scenario in 7 out of 10 participants. Overall, the great majority of participants correctly matched the traffic light (yellow and red) with the simulated case-scenario and appropriately escalated treatment. We found a non-significant 43% reduction in the odds of TI by identifying the red traffic light. We also identified decision fatigue in the control group, but not in the intervention group. This finding suggests that the educational intervention may promote the continuity of accurate therapeutic decisions over time (by ameliorating the impact of decision fatigue on TI) despite the increasing number of case-scenarios.

The use of the TLS is a novel initiative to optimize decisions. It has been successfully applied to different medical fields, including the selection of healthier food choices leading to weight loss or the detection of children with fever at high risk of developing a serious bacterial infection (16, 18). Stangel and colleagues proposed the TLS to monitor treatment response in patients with RRMS. They included a more sophisticated scoring system (0–3) to categorize clinical relapses, evidence of disease progression, a cognitive assessment, and MRI findings. This scoring system leads to a decision model that uses the TLS to facilitate therapeutic choices (38). At the time of writing this manuscript, there were no data available on the application of this strategy.

Our study has some significant limitations. First, the sample size is small given the pilot design. As a consequence, our study was not powered to determine the efficacy of the educational intervention. Second, we used simulated case-scenarios that may not accurately reflect therapeutic decisions in clinical practice or known patients followed up over time. Third, some participants’ responses may reflect local limitations in the prescription of disease modifying agents. Fourth, we only tested some physician-level factors that may influence TI. Finally, we do not know if the educational intervention would require reinforcement months later to maintain its potential effect on TI.

Despite these limitations, our study suggests that a simple educational intervention applying the TLS is feasible to increase clinician’s recognition of MS patients at high risk of progression and overcome decision fatigue. Although our study evaluated therapeutic decisions in MS, the educational intervention could be applied to the management of other medical conditions and thus have wider-reaching implications for clinical care. Furthermore, our results serve as the basis for sample size calculations in the design of future studies.

Increasing awareness is the first step in the decision-making process to reduce the effects of TI. We used the TLS to increase awareness of treatment-relevant knowledge. Our study is also strengthened by: (i) a randomized design, (ii) the application of an evidence-based educational approach following the GREET guidelines (26), (iii) the implementation of a simple educational intervention that links to the neural pathways involved in decision-making under uncertainty (19, 27), and (iv) the target of a clinically relevant outcome (i.e., TI and decision fatigue) (4, 36) with the goal of overcoming knowledge-to-action gaps in MS treatment.

The next steps would include the implementation of studies at a larger scale to determine the efficacy of our educational intervention in overcoming decision fatigue and reducing TI among primary care physicians and specialists managing patients with neurological (MS, stroke) and other chronic conditions (e.g., atrial fibrillation, diabetes).

## Ethics Statement

This study was carried out in accordance with the recommendations of St. Michael’s Hospital Ethical Committee with online informed consent from all subjects. All subjects gave online informed consent in accordance with the Declaration of Helsinki. The protocol was approved by the St. Michael’s Hospital Ethical Committee.

## Author Contributions

GS: study concept and design, creation of the educational intervention, acquisition of data, analysis and interpretation of the data, and obtaining funding. AS: study concept and design, interpretation of the data, and critical revision of the manuscript for intellectual content. MT: study implementation, interpretation of the data, and critical revision of the manuscript for intellectual content. MM: study concept and design, design of the educational intervention, interpretation of the data, and critical revision of the manuscript for intellectual content. CR, JM, and PT: study concept and design, interpretation of the data, critical revision of the manuscript for intellectual content, and study supervision. XM: supervision of MS case-scenarios, interpretation of the data, and critical revision of the manuscript for intellectual content.

## Conflict of Interest Statement

PT and CR were funded by the Swiss National Science Foundation (PNT: PP00P1_150739, CRSII3_141965, and 00014_165884, CCR: 105314_152891, CRSII3_141965, and 320030_143443). GS is supported by the Heart and Stroke Foundation Distinguished Clinician-Scientist and Mid-Career Awards following an open peer-reviewed competition. JM is an employee of Roche Farma Spain. MS, MT, and XM—no disclosures.
