# Supplementary material for: Overcoming Therapeutic Inertia in Multiple Sclerosis Care: A Pilot Randomized Trial Applying the Traffic Light System in Medical Education
Source: Front Neurol. 2017 Aug 21;8:430. doi: 10.3389/fneur.2017.00430 (PMC5567059; doi:10.3389/fneur.2017.00430)
Supplement: Supplementary file 1 [file data_sheet_1.pdf]

Appendix: CASE-VIGNETTES

CASE-VIGNETTES Part I

1. A 42-year old woman diagnosed of RRMS 8 years ago has been under IM interferon (IFN) beta1a treatment. Her last relapse was 3 years ago. She complaints of progressive memory difficulties and lack of attention in the last 12 months. No recent relapses. Her husband confirms the cognitive symptoms affecting her daily activities. No evidence of a mood disorder. EDSS stable. Symbol Digit Modalities Test (SDMT) score of 43 (a score of 55 or lower accurately classified cognitive impairment in MS patients). Current brain MRI: 5 new T2 periventricular lesions, no T1 Gadolinium-enhanced lesions.

What would you do? Please select one:

. Continue on the same DMT and perform a new neuropsychological evaluation in 6 months

. Stop IFN and start her on a monoclonal antibody agent (Natalizumab/Alemtuzumab)

. Stop IFN and start her on Fingolimod

. Stop IFN and start her on Glatiramer acetate

. Stop IFN and start her on Teriflunomide

2. A 27-year old woman with a diagnosis of RRMS has been on SC interferon beta1a (IFN) for 18 months. She had two non-disabling recurrent events since the initiation of IFN. Her EDSS score is 2.0.
A repeated brain MRI revealed 4 new T2 bilateral periventricular lesions and one subcortical Gadolinium-enhanced T1 lesion compared to the MRI prior to the initiation of IFN (4 lesions).

What would you do? Please select one:

. Stop IFN and start her on Teriflunomide

. Stop IFN and start her on Fingolimod.

. Stop IFN and start her on a monoclonal agent (Natalizumab, Alemtuzumab).

. Continue with IFN given the low volume of lesions

. Continue with IFN and reassess in 6 months

3. A 29-year old woman with a diagnosis of RRMS has been on SC interferon beta1a (IFN) for 20 months. She had two recurrent events since the initiation of IFN. Her EDSS score is 2.0.
A control brain MRI revealed 6 new T2 bilateral periventricular lesions and one subcortical Gadolinium-enhanced T1 lesion compared to the MRI prior to the initiation of IFN:

What would you do? Please select one:

. Stop IFN and start her on Teriflunomide.

. Stop IFN and start her on Fingolimod.

. Stop IFN and start him on Peg-interferon β-1a (Plegridy)

Stop IFN and start her on a monoclonal antibody agent (e.g. Natalizumab, etc)

. Continue on IFN and clinical and radiological reassessment in 6-12 months.

Appendix: CASE-VIGNETTES

4. A 40-year old man diagnosed of RRMS 3 years ago (EDSS score of 2) developed a new onset of imbalance and double vision. He had 2 previous relapses (optic neuritis and hypoesthesia in right upper limb). He has been on SC interferon beta 1a since the diagnosis. A new brain MRI showed two T1 Gadolinium-enhanced lesions involving the brainstem and upper cerebellar peduncle.

i) What would you do? Please select one

- Stop IFN and start him on Glatiramer acetate
- Continue on the current treatment
- Stop IFN and start him on a monoclonal antibody (Natalizumab/Alemtuzumab)
- Stop IFN and start him on Teriflunomide
- Stop IFN and start him on Fingolimod
- Stop IFN and start him on Dimethyl fumarate
- Continue on the current treatment and reassessment in 6 months

5. A 25-years old woman developed a facial palsy and headache. Neurological examination showed a right peripheral facial palsy and hypoesthesia on the left upper limb. A MRI showed a T1 Gadolinium-enhancing lesion in the right-pons and 10 T2 periventricular and juxtacortical hyperintense lesions. You treated her with methylprednisolone and then started Interferon B1a treatment. Six months later, she developed loss of strength in the right arm.

What would you do? Please select one.

- Continue Interferon B1a and re-assess in 6 months
- Stop Interferon B1a and start her on Fingolimod
- Stop Interferon B1a and start him on Peg-interferon β-1a (Plegridy)
- Stop Interferon B1a and start her on a monoclonal antibody agent (Natalizumab/Alemtuzumab)
- Stop Interferon B1a and start her on Teriflunomide

6. A 44-year old woman was diagnosed with RRMS 5 years ago. In total, she has had 4 a new relapses. Has been stable on IFN B1a subcutaneous. Her EDSS 3.5. A recent MRI revealed a 10 old periventricular and 1 new lesion on left frontal lobe. There was no GAD enhancing lesions. She has been feeling unwell noticing a decline in her ability to walk over the last 6 months.

What would you do? Please select one

- Continue the treatment with IFN
- Stop IFN and start her on Fingolimod
- Stop IFN and start her on Dimethyl fumarate
- Stop IFN and start her on Teriflunomide
- Stop IFN and start her on a Monoclonal agent (e.g. Natalizumab, Alemtuzumab, etc)
- Continue the treatment with IFN and reassessment in 6 months.

Appendix: CASE-VIGNETTES

7. A 37-year old woman was diagnosed with RRMS 5 years ago. She came to your clinic for a follow-up. In total, she had 3 relapses prior starting on IM interferon (IFN) once weekly. She has been clinically stable in the last 2 years. Her EDSS score is 4. A new brain MRI revealed a new T1 Gadolinium-enhanced peritrigonal lesion (12 mm) in addition to the old confluents periventricular lesions.

What would you do? Please select one

- Continue with the current therapy
- Stop IFN and start her on Fingolimod
- Stop IFN and start her on Dimetyl-fumarate
- Stop IFN and start her on Teriflunomide
- Stop IFN and start her on a monoclonal antibody agent (Natalizumab/Alemtuzumab)
- Continue with the current therapy and reassessment in 6 months

8. A 35-year old man was diagnosed with RRMS 3 years ago. He had 3 relapses while being on Glatiramer. He was switched to Teriflunomide one year ago. Three month ago, he developed an episode of unsteadiness lasting for 10 days. A recent brain MRI revealed a new T1 Gadolinium-enhanced and 4 small new T2 periventricular lesions.

What would you do? Please select one

- Continue with the current therapy
- Stop Teriflunomide and start him on Fingolimod
- Stop Teriflunomide and start him on Peg-interferon β-1a (Plegridy)
- Stop Teriflunomide and start him on a monoclonal antibody agent (Natalizumab/Alemtuzumab)
- Continue with the current therapy and reassess in 6 months.

9. A 22-year old woman was diagnosed with RRMS 5 years ago. She had one relapse while being on Glatiramer in the last two years. Three month ago, she developed an episode of facial numbness and right sided weakness. A brain MRI revealed 5 small new T2 periventricular lesions. None enhanced with gadolinium.

What would you do? Please select one

- Continue with the current therapy and reassess in 6 months.
- Stop Glatiramer and start him on Fingolimod
- Stop Glatiramer and start him on Teriflunomide
- Stop Glatiramer and start him on Peg-interferon β-1a (Plegridy)
- Stop Glatiramer and start him on a monoclonal antibody agent (Natalizumab/Alemtuzumab)
- Continue with Glatiramer

10. A 27-year old woman was diagnosed with RRMS 2 years ago. She had two relapses while being on Interferon SC. She was switched to Dimethyl Fumarate 9 months ago. Recently, she developed an episode of bilateral leg numbness and urinary incontinence that lasted two weeks. A spinal MRI revealed dorsal lesion that enhanced gadolinium at the T10 level.

Appendix: CASE-VIGNETTES

What would you do? Please select one

- Continue with the current therapy and reassess in 6 months.
- Stop Dimethyl Fumarate and start him on Fingolimod
- Stop Dimethyl Fumarate and start him on Teriflunomide
- Stop Dimethyl Fumarate and start him on Peg-interferon β-1a (Plegridy)
- Stop Dimethyl Fumarate and start him on a monoclonal antibody agent (natalizumab/alemtuzumab)
- Continue with Dimethyl Fumarate

CASE-VIGNETTES Part II

11. A 40-year old woman diagnosed of RRMS 5 years ago has been under IM interferon (IFN) beta1a treatment.
Her last relapse was 2 years ago. She noticed progressive memory difficulties and lack of attention in the last 12 months.
No recent relapses. Her husband noticed she has been forgetful affecting her daily activities. She denied depression. EDSS stable. Her Symbol Digit Modalities Test (SDMT) score of 44 (a score of 55 or lower accurately classified cognitive impairment in MS patients).
Current brain MRI: 5 new T2 periventricular lesions.

What would you do? Please select one:

. Continue on the same DMT and perform a new neuropsychological evaluation in 6 months

. Stop IFN and start her on a monoclonal antibody agent (Natalizumab/Alemtuzumab)

. Stop IFN and start her on Fingolimod

. Stop IFN and start her on Glatiramer acetate

. Stop IFN and start her on Teriflunomide

12. A 29-year old woman with a diagnosis of RRMS has been on SC interferon beta1a (IFN) for 18 months. She had two non-disabling recurrent events since the initiation of IFN. Her EDSS score is 2.5.
A repeated brain MRI revealed 4 new T2 bilateral periventricular lesions and one subcortical Gadolinium-enhanced lesion compared to the MRI prior to the initiation of IFN (6 lesions in Flair T2 sequence).

What would you do? Please select one:

. Stop IFN and start her on Teriflunomide

. Stop IFN and start her on Fingolimod.

. Stop IFN and start her on a monoclonal agent (e.g. Natalizumab, Alemtuzumab).

. Continue with IFN given the low volume of lesions

. Stop IFN and start her on peg-interferon B1a (Plegridy)

. Continue with the current therapy and reassess in 6 months.

Appendix: CASE-VIGNETTES

13. A 28-year old woman with a diagnosis of RRMS has been on SC interferon beta1a (IFN) for 16 months. She had two non-disabling recurrent events since the initiation of IFN. Her EDSS score is 1.5.

A control brain MRI revealed 5 new T2 bilateral periventricular lesions and one subcortical Gadolinium-enhanced lesion compared to the MRI prior to the initiation of IFN:

What would you do? Please select one:

. Continue with the current therapy

. Stop IFN and start her on Teriflunomide.

. Stop IFN and start her on Fingolimod.

. Stop IFN and start her on a monoclonal antibody agent (e.g. Natalizumab, Alemtuzumab)

. Stop IFN and start her on Glatiramer

. Continue on IFN and reassess in 6 months.

14. A 38-year old man diagnosed of RRMS 4 years ago (EDSS score of 2.5) developed a new onset of imbalance and double vision. He had 2 previous relapses (optic neuritis and hypoesthesia in right upper limb). He has been on SC interferon beta 1a since the diagnosis. A new brain MRI showed two T1 Gadolinium-enhanced lesions involving the pons and cerebellar peduncle.

i) What would you do? Please select one

- Stop IFN and start him on Glatiramer acetate
- Continue on the current treatment
- Stop IFN and start him on a monoclonal antibody (Natalizumab/Alemtuzumab)
- Stop IFN and start him on Teriflunomide
- Stop IFN and start him on Fingolimod
- Stop IFN and start him on Dimethyl fumarate
- Continue with the current therapy and reassess in 6 months.

15. A 28-years old woman developed facial numbness and mild headaches. Neurological examination showed a subjective diminished sensation on the left face (forehead and cheek) and hypoesthesia on the right upper limb. A MRI showed a T1 Gadolinium-enhancing lesion in the right-pons and 6 T2 periventricular and juxtacortical hyperintense lesions. You treated her with methylprednisolone and then started IFN beta 1A. Six months later, she developed loss of strength in the left arm.

What would you do? Please select one.

- Continue IFN and re-assess in 6 months
- Stop IFN and start her on Fingolimod
- Stop IFN and start her on a monoclonal antibody agent (natalizumab/alemtuzumab)
- Stop IFN and start her on Teriflunomide
- Stop IFN and start her on Glatiramer
- Continue on IFN

Appendix: CASE-VIGNETTES

16. A 43-year old woman was diagnosed with RRMS 6 years ago. In total, she has had 4 a new relapses. Has been stable on IFN B1a subcutaneous. Her EDSS 3.0. She has been feeling unwell noticing fatigue, and generalized weakness in the last 6 months.
A recent MRI revealed a 12 old periventricular and 1 new subcortical lesion on right frontal lobe. There was no GAD-enhancing lesions.

What would you do? Please select one

- Continue the treatment with IFN
- Stop IFN and start her on Fingolimod
- Stop IFN and start her on Dimetyl Fumarate
- Stop IFN and start her on Teriflunomide
- Stop IFN and start her on a Monoclonal agent (e.g. Natalizumab, Alentuzumab, etc)
- Continue with the current therapy and reassess in 6 months.

17. A 33-year old woman was diagnosed with RRMS 4 years ago. She came to your clinic for a follow-up. In total, she had 3 relapses prior starting on IM interferon (IFN) once weekly. She has been clinically stable in the last 2 years. Her EDSS score is 4.5. A new brain MRI revealed a new T1 Gadolinium-enhanced subcortical lesion (14 mm) in addition to the old confluents periventricular lesions.

What would you do? Please select one

- Continue with the current therapy
- Stop IFN and start her on Fingolimod
- Stop IFN and start her on Dimethyl fumarate
- Stop IFN and start her on Teriflunomide
- Stop IFN and start her on a monoclonal antibody agent (natalizumab/alemtuzumab)
- Continue with the current therapy and reassess in 6 months.

18. A 36-year old man was diagnosed with RRMS 2.5 years ago. He had 2 relapses while being on Glatiramer. He was switched to Teriflunomide one year ago. Four months ago, he developed an episode of numbness and weakness on his left side that resolved within 6 days. A recent brain MRI revealed 5 small new T2 periventricular lesions, one of which enhanced with gadolinium.

What would you do? Please select one

- Continue with the current therapy and reassess in 6 months.
- Stop Teriflunomide and start him on Fingolimod
- Stop Teriflunomide and start him on Peg-interferon β-1a (Plegridy)
- Stop Teriflunomide and start him on a monoclonal antibody agent (natalizumab/alemtuzumab)
- Continue on Teriflunomide

Appendix: CASE-VIGNETTES

19. A 25-year old woman was diagnosed with RRMS 4 years ago. She had one relapse while being on Glatiramer two years ago.. Three month ago, she developed an episode of right sided weakness. A brain MRI revealed 6 small new T2 periventricular lesions. None enhanced with gadolinium.

What would you do? Please select one

- ○ Continue with the current therapy and reassess in 6 months.
- ○ Stop Glatiramer and start him on Fingolimod
- ~~○~~ Stop Glatiramer and start him Teriflunomida
- ○ Stop Glatiramer and start him on Peg-interferon β-1a (Plegridy)
- ○ Stop Glatiramer and start him on a monoclonal antibody agent (natalizumab/alemtuzumab)
- ○ Continue with Glatiramer

20. A 28-year old woman was diagnosed with RRMS 2 years ago. She had two relapses while being on Glatiramer. She was switched to Dimethyl Fumarate 10 months ago. Recently, she developed an episode of double vision (left INO) and right sided weakness that resolved in 3 weeks. A brain MRI revealed a 15 mm GAD-enhanced L-paramedian pontine lession.

What would you do? Please select one

- ○ Continue with the current therapy and reassess in 6 months.
- ○ Stop Dimethyl Fumarate and start him on Fingolimod
- ○ Stop Dimethyl Fumarate and start him on Teriflunomide
- ○ Stop Dimethyl Fumarate and start him on Peg-interferon β-1a (Plegridy)
- ○ Stop Dimethyl Fumarate and start him on a monoclonal antibody agent (natalizumab/alemtuzumab)
- ○ Continue with Dimethyl Fumarate

--- END ---

THANK YOU FOR YOUR PARTICIPATION
